# The Effect of Dark Septate Endophytic Fungi on *Mahonia oiwakensis*

**DOI:** 10.3390/plants10081723

**Published:** 2021-08-20

**Authors:** Lei-Chen Lin, Yin-Ling Tan, Wan-Rou Lin, Kuo-Lung Ku, Shang-Tse Ho

**Affiliations:** 1 Department of Forestry and Natural Resources, National Chiayi University, Chiayi 60004, Taiwan; linerm@mail.ncyu.edu.tw (L.-C.L.); kantianiss1790@gmail.com (Y.-L.T.); 2 Bioresource Collection and Research Center (BCRC), Food Industry Research and Development Institute (FIRDI), Hsinchu 30062, Taiwan; wrl@firdi.org.tw; 3 Department of Applied Chemistry, National Chiayi University, Chiayi 60004, Taiwan; 4 Department of Wood Based Materials and Design, National Chiayi University, Chiayi 60004, Taiwan

**Keywords:** berberine, *Cladophialophora chaetospira*, dark septate endophyte, growth response, *Hymenoscyphus*, *Mahonia oiwakensis*

## Abstract

This is the first study to discuss the effects of dark septate endophytes (DSE) on the growth promotion and berberine concentration in *Mahonia oiwakensis*, whose extract (MOE) has been suggested to have potential therapeutic effects against human lung cancer. First, as per phylogenetic analysis, the strains were divided into four groups: CkDB2, CkDB5, MoAL2 and MoAL5. All of these were DSEs, which could form microsclerotia in *M. oiwakensis*. The growth response experiment revealed that inoculation of the plant with MoAL5 and CkDB5 promoted an increase in the total fresh weight of the seedlings. Chemical composition analysis showed that seedlings inoculated with CkDB5 had the highest berberine concentration. These results showed that some DSEs have the ability to promote growth and induce phytochemical responses in the host plant.

## 1. Introduction

The *Mahonia* genus, a member of the Berberidaceae family, contains more than 60 species and is native to Asia and America [1]. Members of this genus include folk medicinal plants [2,3], and *Mahonia* species, in particular, possess antibacterial, antifungal and anti-inflammatory properties [4]. Many isoquinoline alkaloids, such as berberine, oxycanthine and tetrandrine, can be isolated from plants such as *M. aquifolium*. Of these, berberine possesses the best characteristics. Previous studies have shown that berberine can inhibit cell growth and induce apoptosis in several human cancer cell lines [5,6,7,8,9,10]. *M. oiwakensis* Hayata (*Alishan mahonia*) is an endemic species of Taiwan and a well-known folk medicinal plant. Wong et al. (2009) demonstrated that *M. oiwakensis* extract (MOE) inhibits the growth of human lung cancer cells in vitro and in vivo and suggested that it has therapeutic potential against human lung cancer [4].

Various forms of mycorrhizal fungi found in nature play important roles in plant nutrition and nutrient cycling [11]. Mycorrhizae promote host plant growth [12,13] and increase stress tolerance [14,15], thus accelerating the synthesis of secondary metabolites in host plants [16,17,18,19,20,21,22]. Many studies have reported the effects of mycorrhizae on the content of secondary metabolites in plants; however, most of these studies have focused on the effects of arbuscular mycorrhizal fungi (AMF) [16,17,18,20,21], though attention has been paid to dark septate endophytes (DSE) as well [19,22]. DSE can promote the uptake of nutrients (such as C, N, and P) of plants and also help plants against the survival stresses caused by biotic and abiotic factors [23]. Furthermore, only one of the AMF studies is related to berberine [16], and there are no reports about the effects of DSE on berberine production in *M. oiwakensis*. Tan et al. (2016) demonstrated that two root-endophytic fungi, MoAL2 and MoAL5, can associate with *M. oiwakensis* seedlings [24]; however, the benefits for the host plant remain unknown. Among the four strains of root-endophytic fungi maintained in our laboratory, two (CkDB2 and CkDB5) have been identified as DSE and have been shown to promote the growth of the host plant [25]. It is well-known that DSEs can promote the growth and survival of the host plant; however, there is currently no research that is focused on the effects of DSEs on the berberine concentration. To the best of our knowledge, this is the first study to report the effects of DSEs on the growth promotion and berberine concentration in *M. oiwakensis*.

## 2. Results

### 2.1. Molecular Phylogenetic Analysis of the Four Strains

**Figure 1:**
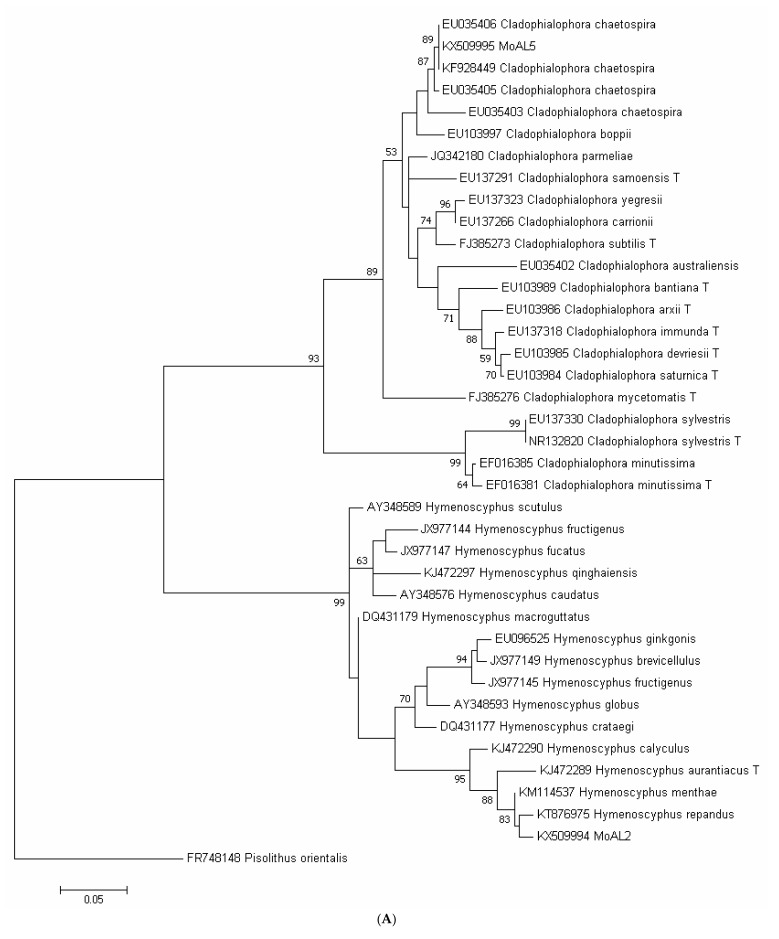

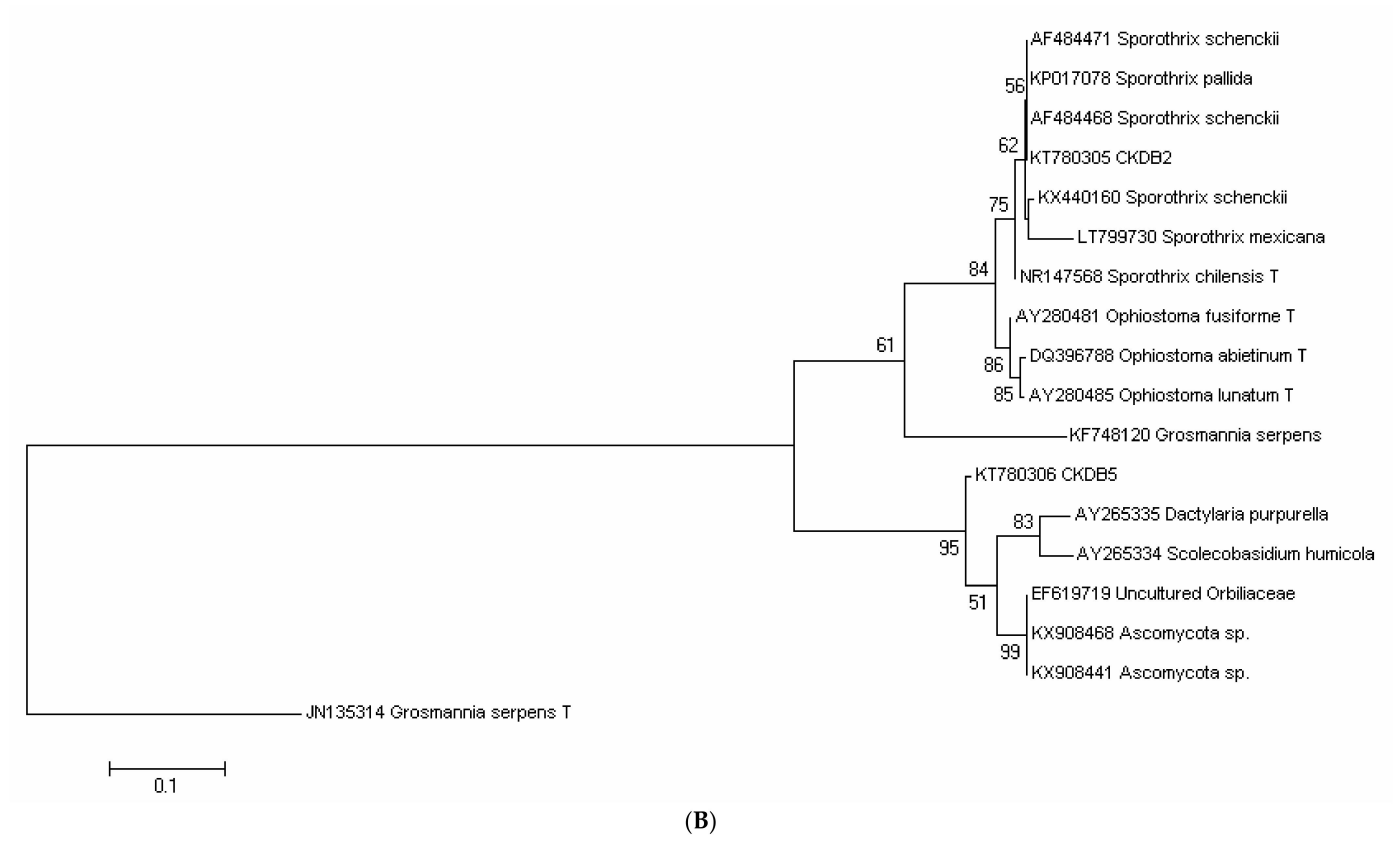
Maximum likelihood phylogenetic tree based on rDNA internal transcribed spacer sequence data from fungal strains MoAL2, MoAL5 (**A**), CkDB2 and CkDB5 (**B**). Bootstrap values greater than 50% are shown above or below branches.

Taxonomic affinities, including the most closely matched sequences, were assigned to MoAL2, MoAL5, CkDB2 and CkDB5 based on BLAST sequence similarity analysis (Figure 1). Through ML (Maximum likelihood) analysis, the ITS (Internal transcribed spacer) sequence of MoAL5 was grouped with sequences of *Cladophialophora* and was found to be closely matched to *C. chaetospira*, with 89% bootstrap values and 100% ITS sequence similarity (Figure 1A). The ITS sequence of MoAL2 closely matched to *Hymenoscyphus repandus* and *H. menthae*, and MoAL2 was identified as a species of *Hymenoscyphus* (Figure 1A). CkDB2 was grouped with sequences of *Sporothrix* and was closely matched to *S. schenckii* (AF484468) (Figure 1B). CkDB5 was grouped with sequences of *Scolecobasidium humicola* and *Dactylaria purpurella* and was closely matched to species of *Ascomycota* (KX908468 and KX908411) with 95% bootstrap values (Figure 1B).

### 2.2. Morphology and Colonization in Resynthesized Seedlings

**Figure 2:**
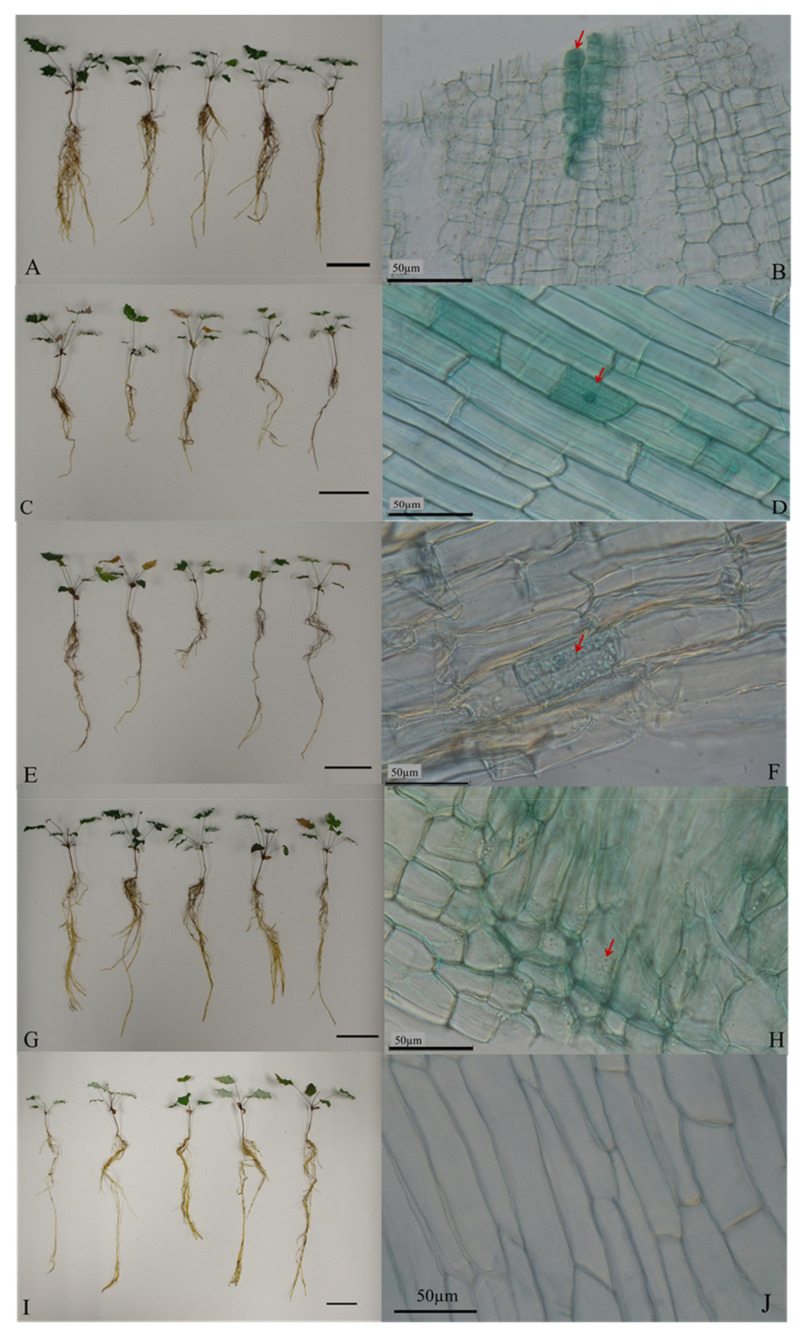
Morphology of *M. oiwakensis* seedlings after incubation for 3 months: (**A**,**C**,**E**,**G**,**I**) show *M. oiwakensis* seedlings of all treatments (bar = 5 cm); (**B**,**D**,**F**,**H**,**J**) show the root stain for all treatments. (**A**,**B**): CkDB2-inoculation; (**C**,**D**): CkDB5-inoculation; (**E**,**F**): MoAL2-inoculation; (**G**,**H**): MoAL5-inoculation; (**I**,**J**): Control. Hyaline microsclerotia-like formations (arrows).

After three months of incubation, all treated seedlings survived (Figure 2A,C,E,G,I). The features of root associations for all treatments were observed by a light microscope (Figure 2B,D,F,H,J). *M. oiwakensi* seedlings inoculated with CkDB2 and CkDB5 grew well (Figure 2A,C), and dark septate microsclerotia-like structures were observed in the stained roots of CkDB2- and CkDB5-treated plants (Figure 2B,D). *M. oiwakensi* seedlings inoculated with MoAL2 and MoAL5 also grew well (Figure 2E,G), and hyaline microsclerotia-like structures were observed in the stained roots of MoAL2- and MoAL5-treated plants (Figure 2F,H). In the controls, the seedlings grew well (Figure 2I); however, no peculiarities were found in the stained roots (Figure 2J).

### 2.3. Growth Responses

As shown in Table 1 and Figure 2A,C,E,G,I, the growth response analyses indicated that not every inoculation had a positive effect on plant growth after incubation for three months. The CkDB5-treated plants’ average shoot fresh weight (ASFW) (0.29 ± 0.13 g), average root fresh weight (ARFW) (0.24 ± 0.09 g), and average total fresh weight (ATFW) (0.53 ± 0.21 g) were significantly different from those of the control groups (*p* < 0.05) and were the highest among all the treated plants [ASFW: MoAL5-treated plants, 0.25 ± 0.07 g; MoAL2-treated plants, 0.15 ± 0.06 g; CkDB2-treated plants, 0.12 ± 0.06 g; and the control, 0.15 ± 0.06; ARFW: MoAL5-treated plants, 0.21 ± 0.07 g; MoAL2-treated plants, 0.10 ± 0.03 g; CkDB2-treated plants, 0.07 ± 0.03 g; and the control, 0.14 ± 0.05 g; ATFW: MoAL5-treated plants, 0.46 ± 0.13 g; MoAL2-treated plants, 0.25 ± 0.09 g; CkDB2-treated plants, 0.19 ± 0.08 g; and the control, 0.29 ± 0.10 g].

### 2.4. Berberine Concentration

**Figure 3:**
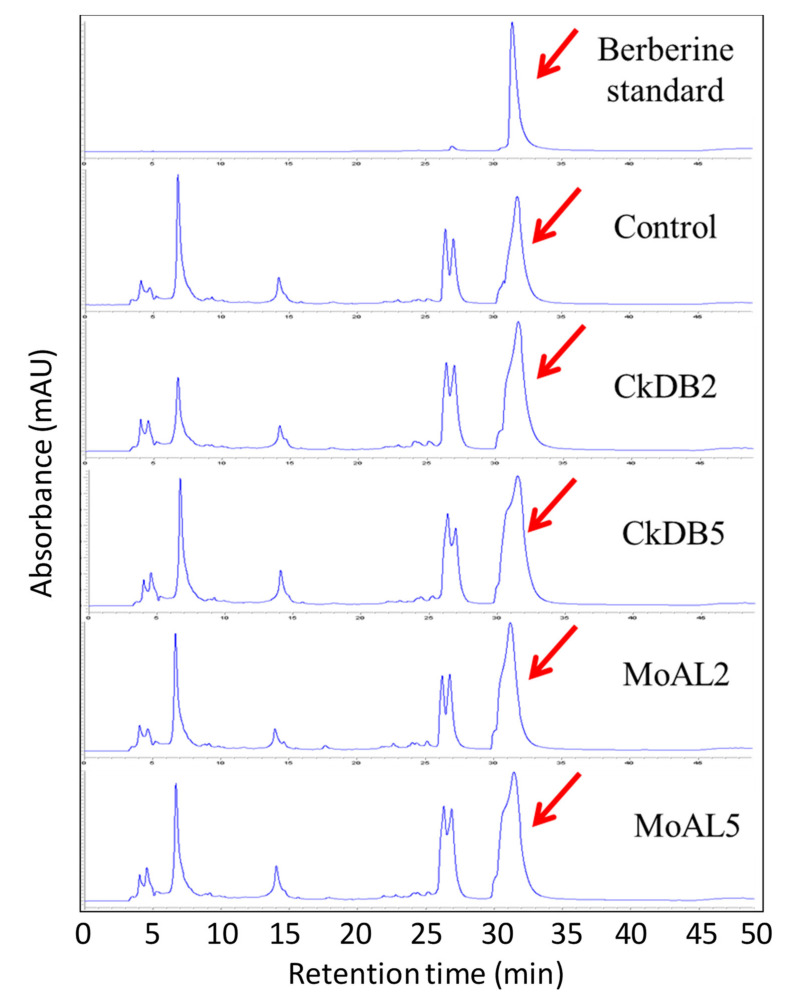
The high-performance liquid chromatography evaluation of berberine production after culture of *M. oiwakensis* seedlings for 3 months in either inoculated or control medium.

The berberine concentrations determined from each treatment were shown to be significantly different from each other (Table 1 and Figure 3). All treated plants had higher berberine concentrations than the controls. The CkDB5-treated plants had the highest berberine concentration (4441 ± 21 µg/g) compared with the other treated plants (MoAL5-treated plants, 3809 ± 144 µg/g; CkDB2-treated plants 3140 ± 176 µg/g; MoAL2-treated plants, 2890 ± 107 µg/g; and the control, 2419 ± 94 µg/g).

## 3. Discussion

Although numerous sterile DSEs have been isolated from different plant roots, they have not been identified at the species level due to their inability to form teleomorphs and conidia [26,27]. However, in recent years, the ITS of rDNA has been successfully used to clarify the phylogenetic relationships and demonstrate the genetic diversity among DSEs [28,29,30,31]. According to the ITS analysis, CkDB2 was a newly recorded species (*S. schenckii*) from Taiwan. *S. schenckii* is distributed throughout the world and causes Sporotrichosis [32]. In our previous study, CkDB2 had no negative effect on its host plants, such as *C. kanehirae* [25] and *M. oiwakensis* (Table 1). The colony and hypha of the CkDB2 [33] showed features that were different from those of other strains of *S. schenckii* [34,35]. Meriden et al. [36] reported that all members of *Ochroconis*, formerly known as *Dactylaria*, are dematiaceous fungi that cause phaeohyphomycosis [37,38,39,40]. Although CkDB5 was found to be closely matched to the *Dactylaria* genus, it can confer positive effects on its host plant (e.g., *C. kanehirae* [25] and *M. oiwakensis*) (Table 1).

However, Figure 1 also indicates that MoAL5 was closely matched to *C. chaetospira* (EU035406). In our previous observations of colony and hypha [24], MoAL5 showed features that were same as that of *C. chaetospira* [41], and MoAL2 had hypha. Thus, MoAL5 can be considered as a newly recorded species (*C. chaetospira*) from Taiwan, whereas MoAL2 may be a new species of the genus *Hymenoscyphus*. Although *Heteroconium chaetospira* (Grove) M.B. Ellis (syn. *C. chaetospira*) is a DSE [42,43], it can colonize the roots of Chinese cabbage without causing any apparent pathogenic symptoms [44,45]. In this study, MoAL5 was found to belong to *C. chaetospira*. It did not cause any apparent pathogenic symptoms in *M. oiwakensis*; in contrast, it promoted the growth of *M. oiwakensis* and facilitated increased production of berberine. One of the features of DSE is hyaline microsclerotia [46,47]. In this study, staining of the roots revealed microsclerotia in the roots of all treated plants. Hence, based on the above results, there is sufficient evidence to demonstrate that CkDB2, CkDB5, MoAL2 and MoAL5 can be classified as DSEs and could associate with *M. oiwakensis*.

Mycorrhizal fungi, DSEs and plant growth-promoting rhizobacteria (PGPR) can promote the growth response of their host plants [42,48,49,50]. DSEs are ascomycetes that can facilitate the growth of their host plants without causing pathologies [42]. CkDB2 and CkDB5 have been previously demonstrated to be DSEs and can promote the growth response in *C. kanehirae* [25]. In this study, CkDB2 and CkDB5 could act as a DSE with *M. oiwakensis* and promote increased production of berberine in *M. oiwakensis* compared with that in the controls. Between these two strains, CkDB5 was able to induce *M. oiwakensis* in obtaining the highest value of fresh weight and could also induce the production of the highest amount of berberine (1.83-fold higher than that of controls). Of the other two strains (MoAL2 and MoAL5), MoAL5 was found to belong to *C. chaetospira*. However, MoAL5 is different from the more common DSEs [51,52,53], which have no significant effects on the fresh weight on plants such as the blueberry plant (*Vaccinium corymbosum* L.) [54]. As reported previously, several arbuscular mycorrhiza fungi were also able to promote berberine contents in *Phellodendron chinense*. For instance, the berberine contents in the root of *P. chinense* seedlings were elevated 1.89-fold compared to control group after inoculated with *Glomus etunicatum* for 3 months [16]. For the first time, our results demonstrated that DSEs were able to promote both of growth performance and berberine production in *M. oiwakensis*.

Colonization by fungi can cause a series of resistance reactions in host plants, including eliciting an effect on secondary metabolites such as alkaloids and terpenoids. [55,56,57]. AMF have been demonstrated to affect the types and concentrations of secondary metabolites [58,59,60], and every species of AMF has a different effect on plants. For example, *G. diaphanum*, *G. etunicatum*, *G. intraradices*, *Acaulospora mellea* and *A. laevis* promote the increased production of camptothecin in *Camptotheca acuminate* [33,59,60], whereas *G. manihot* reduces it [35]. Furthermore, Zhou & Fan [16] have also shown that AMF can not only promote growth but also increase the berberine content in *P. chinense*. Some DSEs are also able to promote the increased production of flavonoids in *S. involucrata* seedlings [19]. In this study, all four strains could facilitate increased production of berberine in *M. oiwakensis*. On the other hand, some endophytic fungi (*Alternaria* sp. and *Fusarium solani*) have been reported with the ability to produce berberine [61,62]. However, how DSE regulate the berberine production in *M. oiwakensis* and its mechanism of action still need to investigate in the future study. Among these four strains, CkDB5 (isolated from *C. kanehirae*) has been demonstrated to have significant effects on the growth of *C. kanehirae*, particularly with respect to root growth [25]. Additionally, based on our results, CkDB5 had similar effects on *M. oiwakensis*. Therefore, it is safe to assume that CkDB5 has wide host-range compatibility and functional diversity.

## 4. Materials and Methods

### 4.1. Seeds

The seeds of *M. oiwakensis* were collected from the Alishan Recreational Park, Alishan Township, Chiayi County, Taiwan (120°48′45″ E, 23°30′46″ N, 2279 m altitude).

### 4.2. Strains

**Figure 4:**
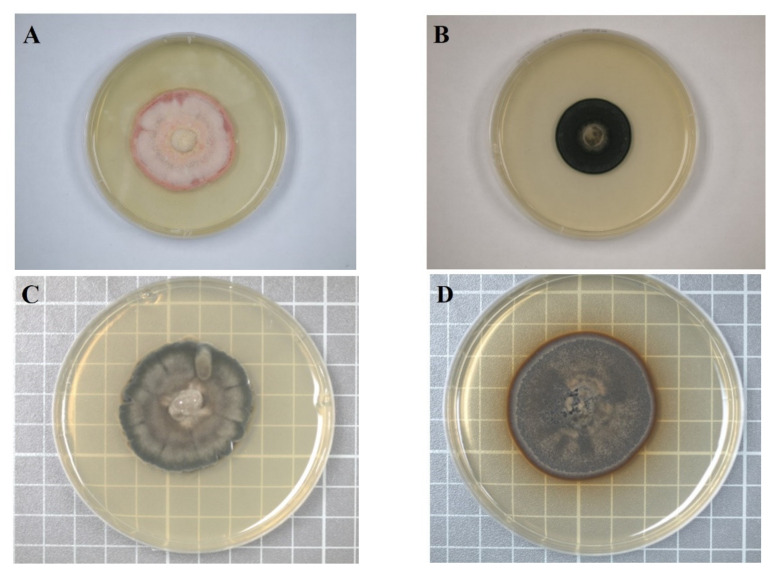
Colony morphology of these four strains on PDA medium. (**A**): MoAL2; (**B**): MoAL5; (**C**): CkDB2; (**D**): CkDB5.

Four strains of DSEs were used in this study. Among these strains, MoAL2 and MoAL5 were isolated from *M. oiwakensis* [24], whereas CkDB2 and CkDB5 were isolated from *Cinnamomum kanehirae* [33]. These four strains were deposited at the Tree Mycorrhiza Laboratory of National Chiayi University. The internal transcribed spacer (ITS) genomic sequences of these four endophytes have been uploaded to GenBank (strains MoAL2 (Figure 4A), MoAL5 (Figure 4B), CkDB2 (Figure 4C) and CkDB5 (Figure 4D): KX509994, KX509995, KT780305 and KT780306, respectively).

### 4.3. DNA Extraction, Sequencing and Phylogenetic Analysis

The fungal mycelium growing on the surface of malt extract agar were subjected to DNA extraction; the obtained DNA was then amplified and sequenced [63,64]. Isolation of genomic DNA was performed using the NucleoSpin^®^ Plant II Kit (MACHERY-NAGEL GmbH & Co. KG, Düren, Germany). The fungal DNA was used as a template for amplification with primers V9G and LR1 [65,66]. The PCR conditions were as follows: 95 °C for 10 min, followed by 35 cycles at 94 °C for 30 s, 50 °C for 40 s and 72 °C for 40 s, with a final extension step at 72 °C for 10 min. The PCR products were analyzed by gel electrophoresis and sequenced by Tri-I Biotech, Inc. Internal transcribed spacer (ITS) sequences were subjected to NCBI MEGABLAST queries. Phylogenetic analysis was performed with MEGA 7.0 for maximum likelihood (ML) analysis based on the ITS sequences [67].

### 4.4. Inoculation with Endophytes

Inoculation was performed using the method of Ann, Tsai, Wang, & Hsien [68] and Zhang, Tang, Chen, & Wang [69] with some modifications. After cleaning, the seeds of *M. oiwakensis* were sterilized with 35% H_2_O_2_ for 3 min and rinsed three times with sterilized distilled water. The seeds were then transferred to a test pot containing a mixture of peat and vermiculite (1:1 *v*/*v*; previously sterilized at 121 °C for 60 min) for germination. The germinated seedlings were then transplanted to new tubes (4 cm in diameter, 18 cm in height) containing a mixture of peat and vermiculite (3:1 *v*/*v*; previously sterilized at 121 °C for 60 min) and were inoculated with the inoculum. These four strains grow on the PDA medium after 21-day incubation, and take out the edge of the colony for inoculum. For inoculation, each seedling was inoculated with two 5-mm diameter pieces of mycelium. Five treatments (one control and four inoculations) were used. Each treatment had five replicates. Each replicate was grown, watered and fertilized in the growth chamber (23 °C, 65% RH and 16:8-h light/dark cycle with 5000 lx as maximum illumination). This method is a kind of mycorrhizal synthesis that can avoid being affected by environmental microorganisms. After three-month incubations, the features of root associations for all treatments were observed using the method of staining root [15].

### 4.5. Plant Growth Responses

To measure the effects of these DSEs on the growth of the seedlings, the seedlings were carefully removed from their substrates after the incubation period, and their fresh weights were measured.

### 4.6. Determination of Berberine Concentrations

All seedlings were sliced into small pieces, air dried in a desiccator with silica gel to constant weight and then pulverized. For each sample, 2.5 mL of methanol was added to an aliquot of 250 μg powder. After 24 h, the mixture was filtered. The residue was rinsed twice with 1.0 mL methanol and pooled with the filter. The pooled solution then was subjected to a 3 mL-methanol, 3 mL-deionized-water pre-conditioned C18 solid-phase extraction cartridge (SPE, Strata C18-E, 55 µm, 70 A) and then flushed with 3 mL of 70% methanol. The eluents were collected and diluted with methanol to exactly 5.0 mL. An aliquot of 20 μL was injected into a high-performance liquid chromatography (HPLC) system for berberine determination. The conditions of HPLC used in the present study were described as below. In brief, the sample was separated by a reversed phase column (4.6 × 250 mm, 5 μm, Discovery C18 HPLC Column, Supelco) with a mobile phase flow rate of 0.8/mL/min, and the eluents were monitored at 260 nm by a UV detector (Agilent HPLC 1100 series, HP). The mobile phase comprised solvent A (methanol: CH₃CN, 1:4) and solvent B (2.5 mM CH₃COONH₄) and was ramped linearly from 10% A/90% B (0 min) to 20% A/80% B (15 min), 30% A/70% B (20 min), 35% A/65% B (40 min) and finally to 100% A/0% B (50 min). A calibration curve (y = 90.124; x−108.16, where y denotes the area signal of 260 nm and x denotes the berberine concentration in μg/mL), was prepared by six point-standard berberine solutions ranging in concentrations from 25 to 250 μg/mL. A correlation coefficient R^2^ = 0.9984 was used to quantify the berberine concentration in the samples.

### 4.7. Statistical Analysis

Statistical Package for the Social Science (SPSS 12.0) (Chicago, IL, USA) for Windows was used to perform all statistical analyses. Tukey’s multiple range test at a significance level of *p* ≤ 0.05 was used to analyze the differences among treatments.

## 5. Conclusions

This study demonstrated that CkDB2, CkDB5, MoAL2 and MoAL5 can associate with the roots of *M. oiwakensis*. Molecular analysis revealed that these four strains should be classified into four groups: CkDB2 belongs to *S. schenckii*; CkDB5 is a member of the Ascomycota; MoAL2 is a member of the genus *Hymenoscyphus*; and MaAL5 belongs to the *C. chaetospira.* Among these four strains, CkDB5 and MoAL2 are newly identified species globally, whereas CkDB2 and MoAL5 are newly identified species in Taiwan. MoAL5 and CkDB5 can help promote growth and increase the production of berberine in *M. oiwakensis*. With CkDB5 inoculation, berberine concentration can be increased by nearly 80%, so CkDB5 can potentially be used to increase the berberine concentration and promote the growth of *M. oiwakensis*.

## Figures and Tables

**Table 1:** Growth and berberine concentrations in *M. oiwakensis* seedlings inoculated with different fungal isolates after three months of incubation.

Treatment	Fresh Weight/g			Berberine Concentration/µg g^−1^
	Shoot	Root	Total	
Control	0.15 ± 0.06 ^bc^	0.14 ± 0.05 ^b^	0.29 ± 0.10 ^bc^	2419 ± 94 ^d^
CkDB2	0.12 ± 0.06 ^c^	0.07 ± 0.03 ^b^	0.19 ± 0.08 ^c^	3140 ± 176 ^c^
CkDB5	0.29 ± 0.13 ^a^	0.24 ± 0.09 ^a^	0.53 ± 0.21 ^a^	4441 ± 21 ^a^
MoAL2	0.15 ± 0.06 ^bc^	0.10 ± 0.03 ^b^	0.25 ± 0.09 ^c^	2890 ± 107 ^c^
MoAL5	0.25 ± 0.07 ^ab^	0.21 ± 0.07 ^a^	0.46 ± 0.13 ^ab^	3809 ± 144 ^b^

All values are means ± standard deviation of five replicates. Values in the same column with different letters (^a^, ^b^, ^c^, etc.) are different at 5% significance level.

## Data Availability

Data is contained within the article.
